# Gut feelings and sweet teeth: nutritional solutions to the gut damage in inflammatory bowel disease and HIV infection

**DOI:** 10.3389/fnut.2026.1878885

**Published:** 2026-07-14

**Authors:** Anna Labiner, Elena-Lia Spoiala, Cristian Apetrei, Laura Mihaela Trandafir, Ivona Pandrea

**Affiliations:** 1 Division of Infectious Diseases, Department of Medicine, School of Medicine, University of Pittsburgh, Pittsburgh, PA, United States; 2 Grigore T. Popa University of Medicine and Pharmacy, Iasi, Romania; 3 Department of Infectious Diseases and Microbiology, School of Public Health, University of Pittsburgh, Pittsburgh, PA, United States; 4 Division of Experimental Pathology, Department of Pathology, Department of Medicine, School of Medicine, University of Pittsburgh, Pittsburgh, PA, United States

**Keywords:** acquired immunodeficiency syndrome (AIDS), diet, gastrointestinal tract, human immunodeficiency virus (HIV), inflammatory bowel disease (IBD), metabolic disease, simian immunodeficiency virus (SIV), tight junctions

## Abstract

Damage to the mucosal layer of the gut is a central feature of chronic inflammatory diseases, often resulting in gut dysbiosis, microbial imbalances, and dysregulated immunometabolism. Gut dysfunction significantly impacts human health, disrupting metabolic, cardiovascular, and neurological systems, and leading to metabolic syndrome. Here, we discuss two clinical entities that are apparently unrelated but actually share similar alterations to gut mucosal barrier integrity: inflammatory bowel disease (IBD) and human immunodeficiency virus (HIV). We use the particular cases of pediatric IBD and nonhuman primate models of HIV infection to compare and contrast the impact of these diseases on gut integrity and microbiome composition. We also explore potential therapies, focusing on dietary interventions. Both HIV infection and IBD cause gut barrier disruption, but via different mechanisms. HIV rapidly depletes mucosal CD4^+^ T cells (especially Th17 cells), weakening epithelial defenses, leading to a loss of tight junctions (“leaky gut”), microbial translocation, and systemic inflammation. In IBD (Crohn’s disease and ulcerative colitis), chronic immune attacks on the gut lining produce ulcers and tight-junction defects. These ulcerations trigger immune-cell infiltration and markedly increase permeability. In both conditions, impaired mucus and epithelial integrity result in higher circulating lipopolysaccharides (LPS), macrophage activation, and systemic inflammation. HIV and IBD also induce distinct yet overlapping dysbioses. HIV infection is associated with markedly reduced bacterial diversity and an overgrowth of potentially inflammatory taxa (e.g., Proteobacteria, Prevotella). Similarly, IBD patients have low diversity and loss of beneficial Firmicutes (notably *Faecalibacterium prausnitzii*) with relative *Proteobacteria* overabundance. In both diseases, the gut flora shifts away from fiber-fermenting commensals to “pathobionts,” fueling local inflammation. While the therapeutic potential of targeting metabolic products is widely explored, there is also a push towards discovering nondrug solutions, particularly through diet and nutrition. We present the effects of micronutrient intake, feeding mechanisms (exclusive enteral nutrition), and different diets (high fiber, Mediterranean, high fat) on disease progression and cellular metabolism. As a low-intervention approach, nutrition has enormous potential to improve human health by reducing inflammation and associated metabolic disturbances. Finally, we emphasize the capabilities of using animal models to elucidate the complexities of disease mechanics in IBD and HIV.

## 1 Introduction

Inflammation is an essential aspect of the body’s defense mechanism, as it fights harmful pathogens and initiates the healing process. While acute inflammation is generally protective and beneficial in the healing process, chronic, low-grade inflammation can contribute to the pathology of a wide variety of conditions. In recent years, 50% of all deaths worldwide were attributable to diseases associated with chronic inflammation, including ischemic heart disease, stroke, cancer, diabetes mellitus, chronic kidney disease, non-alcoholic fatty liver disease, and autoimmune and neurodegenerative conditions (1). In addition to chronic infections, smoking, diet, sedentary lifestyle, obesity, hormones, stress, and irregular sleep patterns are all contributors to low-level chronic inflammation (2).

The composition of the gut microbiota is similarly regarded as a contributing factor to chronic inflammation. Given this, recent studies have focused on the relationship between immune activation and gut dysfunction. Damage to the mucosal layer of the gut is a central feature of multiple chronic inflammatory illnesses like IBD, such as Crohn’s disease (CD) or ulcerative colitis (UC), as well as HIV, SARS-CoV-2, rheumatoid arthritis, neurodegenerative disorders, and metabolic syndrome (MetS), among others (3–7). Often resulting in gut dysbiosis, microbial imbalances, and dysregulated immunometabolism, damage to the mucosal layer can prompt further inflammation and immune activation, creating a cycle that exacerbates disease symptoms (6).

**Figure 1:**
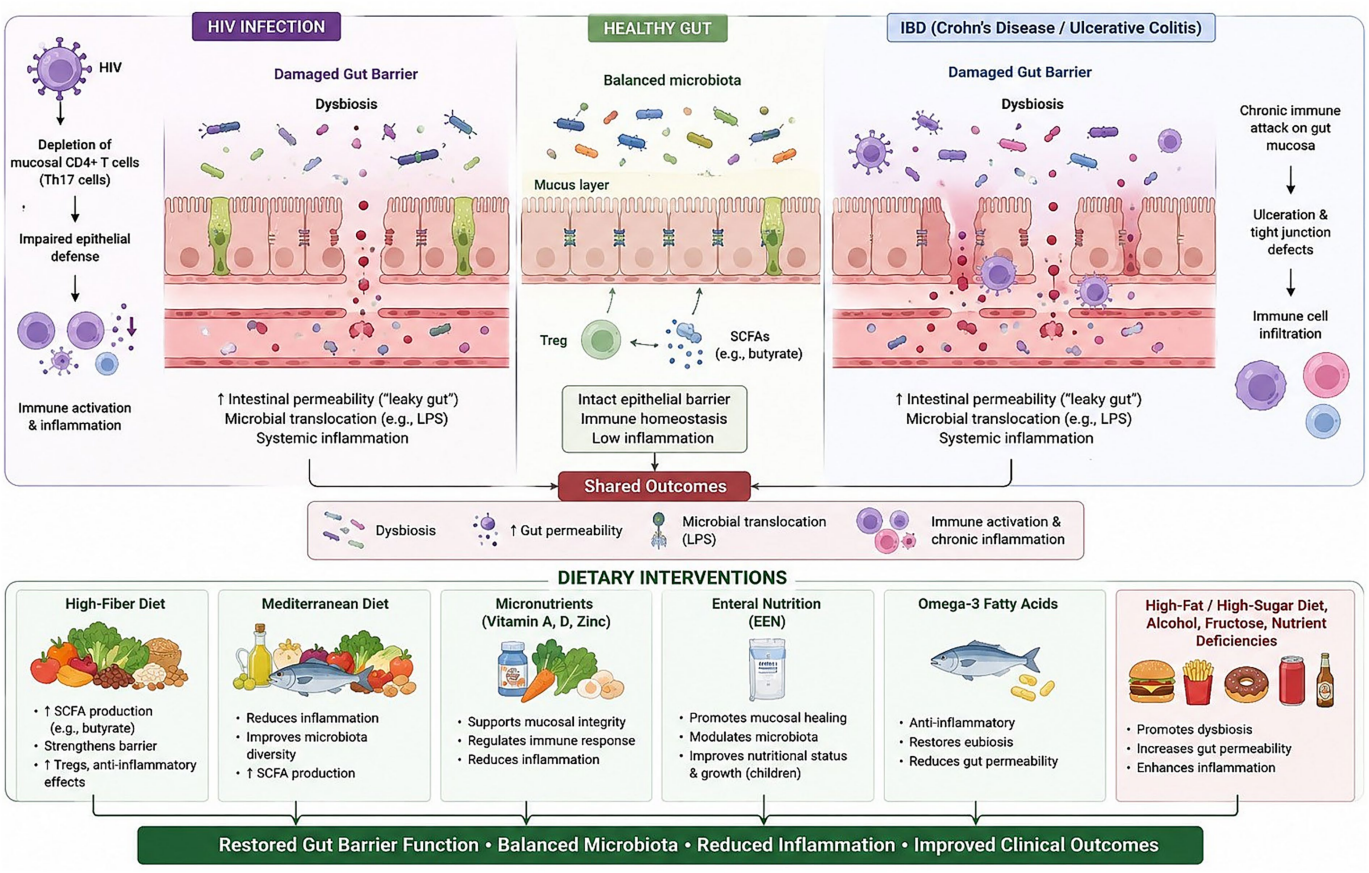
Gut barrier dysfunction and microbiota alterations in human/simian immunodeficiency virus (HIV/SIV) infections and inflammatory bowel disease (IBD). Dietary interventions can significantly contribute to the clinical management of both conditions through mucosal barrier restoration, microbiome switches toward non-inflammatory phenotypes, and control of mucosal and systemic inflammation.

Here, we will use HIV and IBD as models of inflammatory disease to assess the immunomodulatory and metabolic effects of gut barrier dysfunction and microbiome alterations (Figure 1).

While the therapeutic potential of targeting the immune system, reducing inflammatory markers, and restoring normal tissue function is being explored, there is also a push towards discovering non-drug solutions, particularly through diet and nutrition (2). Therefore, we will analyze the effects of micronutrient intake, feeding mechanisms (exclusive enteral nutrition), and different diets (high fiber, Mediterranean, high-fat) on disease progression and cellular metabolism. As a low-intervention approach, nutrition has enormous potential to improve human health by reducing inflammation and associated metabolic disturbances.

## 2 Methodology

This is a narrative review aiming to synthesize and critically discuss current evidence on the impact of gut mucosal barrier dysfunction, microbiota alterations, immunometabolic changes, and corrective dietary/nutritional interventions in IBD and HIV infection. We conducted a targeted literature search between January 2000 and March 2026 across the PubMed, StatPearls, ScienceDirect, Scopus, Web of Science, Wiley Online Library, and Oxford Direct databases, using the following keywords and/or their combinations: “HIV,” “AIDS,” “inflammatory bowel disease,” “Crohn’s disease,” “ulcerative colitis,” “gut microbiota,” “intestinal permeability,” “leaky gut,” “mucosal barrier,” “microbial translocation,” “diet,” “nutrition,” “Mediterranean diet,” “fiber,” “total parenteral nutrition,” “omega-3 fatty acids,” “vitamin A,” “PPARγ,” “fecal microbiota transplantation,” and “SIV models.”

Only peer-reviewed articles written in English were selected and compiled into an exhaustive library using the EndNote software. In the library we did not include any article from predatory journals. From the library, we selected original studies and reviews published between 2002 and 2026.

We selected recent position papers addressing IBD classification and global HIV/AIDS management guidelines, nutritional recommendations for chronic disease, and microbiome-targeted therapeutic strategies. Also, we selected international reports available from major national and international agencies. When web sites were used, we selected only governmental sites (i.e., Centers for Diseases Control and Prevention-CDC) and sites of international organizations (i.e., World Health Organization-WHO). These sites were also used as sources for national and international policies.

Since this is a narrative review, no quantitative meta-analyses or formal quality appraisal were performed. To analyse the available data, we integrated it into the larger themes of our study to show statistical trends, effects of previously tested dietary or drug interventions, and future directions for research. This evidence was compiled with the goal of clearly and concisely presenting the data in a way that is easy to absorb by a broad readership, with an elementary background in paediatrics and infectious diseases.

## 3 Definitions, classifications, and pathogenesis

### 3.1 Inflammatory bowel disease (IBD)

IBD is an umbrella term for a wide spectrum of disorders marked by persistent gastrointestinal inflammation of multifactorial and not yet fully elucidated origin, typically following a relapsing–remitting course (8). The principal phenotypes are CD and UC, with a smaller subset of patients being classified as having IBD-unclassified (IBD-U). In children, very-early-onset IBD (VEO-IBD) is defined as a disease with onset at 6 years of age or younger, and represents a distinct clinical entity, accounting for approximately 6–15% of childhood IBD cases (9).

To standardize age-based classification, pediatric IBD is categorized based on several international schemes. The Montreal classification defines pediatric-onset disease (A1) as diagnosis before 17 years of age (10). A1 pediatric-onset disease is further subdivided into A1a (diagnosis before 10 years) and A1b (10–17 years) by the Paris classification, developed by the ESPGHAN Porto group (11). Within the A1a subgroup, children diagnosed before age 6 are considered to have VEO-IBD, with further distinctions for infantile-onset (≤2 years) and neonatal-onset (≤28 days) disease (11).

Particularly concerning is the rising incidence of IBD among very young children (aged <6 years), a population that frequently presents with more severe and treatment-refractory disease phenotypes (12). When compared with the later-onset pediatric IBD, VEO-IBD is more frequently associated with single-gene defects, immune dysregulation, severe disease course, and refractoriness to conventional therapies, underscoring the need for an early diagnostic and targeted evaluation (12). Unlike adult-onset IBD, which results from a complex interaction between environmental exposures, microbiota alterations, and polygenic susceptibility, VEO-IBD generally has a strong monogenic basis (13). More than 80 monogenic disorders have been associated with intestinal inflammation resembling CD or UC (14). These defects primarily affect epithelial barrier integrity, microbial sensing, immune regulation, and lymphocyte function (15). Genes implicated in VEO-IBD can be broadly grouped into several categories based on their pathogenic impact, being responsible for: epithelial barrier defects (i.e., TTC7A, COL7A1, FERMT1), innate immune and antimicrobial function defects (i.e., CYBB, NCF1, NCF2), IL-10 signalling defects (IL10, IL10RA, IL10RB), regulatory T-cell dysfunction (FOXP3; IPEX syndrome), T- and B-cell immunodeficiencies (RAG1, RAG2, DOCK8, LRBA, CTLA4), and inflammasome or autoinflammatory pathways (NLRC4, MEFV, PLCG2) (16). Although NOD2 is strongly associated with CD susceptibility, it is more commonly implicated in polygenic forms rather than classical monogenic VEO-IBD (17). Among these pathways, defects in IL-10 signalling represent one of the best-characterized causes of severe infantile colitis, often presenting with early-onset, treatment-refractory disease (18).

The analysis of the Global Burden of Inflammatory Bowel Disease 2021 reported that, while the overall global incidence of IBD in children and adolescents (0–19 years) remained largely stable between 1990 and 2021, substantial geographic variation persisted, with decreasing or plateauing rates in high-income regions such as North America and Western Europe, and significant increases in rapidly industrializing regions, particularly East Asia (19).

The burden of pediatric IBD extends beyond somatic manifestations, as chronic gut inflammation often leads to malnutrition, impaired linear growth, and increased psychosocial stress (20). Pediatric IBD is generally more extensive and severe than adult-onset IBD. In ulcerative colitis, pancolitis is the rule: over 85% of childhood-onset UC involves the entire colon (Paris E3/E4), far higher than in adults (21). Young children with CD also tend to have colonic-predominant disease. In a study including 1,092 children with IBD, colon-only IBD (either UC/IBD-U or CD limited to colon) was present in 81% of children <6 and 65% of those 6–10 years old; distal ileal involvement increased with age (21). Deep ulcerations, fistulas, and strictures accumulate rapidly: by diagnosis, ≥70% of pediatric patients have moderate-to-severe activity (21). These aggressive features (panintestinal lesions, growth failure) necessitate prompt, multidisciplinary management. Importantly, pediatric IBD represents a particularly relevant model for studying early nutritional and microbiome-targeted interventions. Because disease onset occurs during critical periods of immune maturation, intestinal microbiome development, and physical growth, uncontrolled inflammation may result in long-term consequences extending beyond the gastrointestinal tract (22). Early therapeutic intervention in children may therefore have a greater capacity to alter disease trajectory, prevent irreversible bowel damage, preserve growth and pubertal development, and promote sustained mucosal healing compared with interventions initiated later in adulthood (23). This concept has contributed to a growing emphasis on early disease control and nutrition-centered therapeutic strategies in pediatric IBD, particularly given the heightened regenerative potential of the developing intestine and microbiome (24). Growth impairment is one of the key extraintestinal issues in pediatric IBD. At the time of diagnosis, 15–40% of children with CD exhibit growth failure, as reflected by reduced height velocity, compared with only 3–10% of pediatric patients with UC (25). If uncontrolled, chronic inflammation and malnutrition can further slow growth and delay puberty (26). These challenges, combined with managing a chronic, lifelong condition, place a substantial psychological burden on affected youth: children and adolescents with IBD experience markedly reduced quality of life, with studies documenting increased rates of depression, anxiety, social isolation, impaired peer relationships, and decreased academic performance among affected children and adolescents (27). Furthermore, because pediatric patients are expected to live with IBD for decades, reducing cumulative inflammatory burden early in the disease course may substantially improve long-term metabolic, developmental, and psychosocial outcomes.

### 3.2 Human immunodeficiency virus (HIV)

HIV is a retrovirus that specifically targets and depletes CD4^+^ T cells, leaving hosts severely immunocompromised. HIV damages certain tissues and organs, including the gut, brain, lungs, liver, heart, and central nervous system (28). Typically, the natural history of HIV infection is comprised of three main stages: (a) acute infection, which is characterized by rapid viral replication and flu-like symptoms. During this stage, the virus rapidly spreads to the lymphoid and mucosal tissues that harbor the HIV hosts, i.e., cells that express the surface receptor CD4 and the virus coreceptor CCR5. These cells are swiftly depleted during the acute infection, and their destruction releases multiple catabolites that induce local mucosal inflammation and enteropathy, causing a leaky gut syndrome; (b) the chronic or asymptomatic stage of infection follows, and it is due to a partial, albeit ineffective, control of viral replication by the host immune response. As a result, the virus multiplies at lower, but detectable levels, which triggers chronic immune activation and inflammation, which are the main causes of disease progression and death in both persons living with HIV (PLWH) and nonhuman primates infected with the simian counterpart of HIV, the simian immunodeficiency virus (SIV) (29–31). Most hosts at this stage of infection remain unaffected clinically; (c) finally, if left untreated or treated inadequately, HIV infection can progress to acquired immunodeficiency syndrome (AIDS), in which viral replication accelerates, inflammation, T cell activation, and coagulation are enhanced, and CD4^+^ T cells are massively depleted, leaving the host vulnerable to opportunistic infections and cancers (32, 33).

HIV and AIDS have posed a major risk to public health on a global scale. However, with the development of antiretroviral therapy (ART) and other intervention techniques, individuals living with HIV/AIDS now have much longer life expectancies, with the disease transforming from a fatal diagnosis to a manageable, chronic illness with a nearly normal lifespan (34). Globally, the incidence of HIV/AIDS decreased by 18.10% from 1990 to 2021, though the burden of the disease varies widely, with two-thirds of global HIV infections concentrated in sub-Saharan Africa (35).

Given the increasing number of individuals aging with HIV, treatment has largely shifted to focus on the effects of long-term antiretroviral therapy usage and managing non-AIDS comorbidities. PLWH are more at risk of developing comorbidities associated with inflammation, including cardiovascular disease, cancer, and kidney, liver, bone, and neurocognitive disease (36). The side effects of long-term ART usage have not been fully uncovered, but the long-term toxicity of the drug is associated with many adverse effects, such as fracture risk and osteoporosis, renal and metabolic disorders, central nervous system (CNS) disorders, cardiovascular disease, and liver disease (37).

Another major focus for research and treatment is the impact of HIV infection on the gut. The gut mucosa contains a significant amount of lymphoid tissue and CD4^+^ T cells, making it an early site of HIV replication and a major reservoir of latent infection. During HIV infection, the gut’s microbial, mechanical, and immunologic barriers are disrupted, promoting chronic immune activation and driving viral persistence (38). This loss of intestinal integrity also results in microbial translocation, which can cause disease progression, early onset of non-AIDS comorbidities, and poor CD4^+^ T-cell recovery on ART (39). While ART is effective at preventing viral replication in peripheral circulation, it does not fully restore the gut microbiome, nor does it eliminate the reservoirs of latent HIV infection (38, 40). In fact, ART may promote further gut dysbiosis in people living with HIV (41). In addition, ART may trigger immune reconstitution inflammatory syndrome (IRIS), a complex disease involving an inflammatory response against underlying opportunistic infections that occurs after initiation of ART as the immune system is reconstituted. Complications of IRIS can be severe, including respiratory failure, CNS complications, progressive multifocal leukoencephalopathy, malignancies, and blindness, among others (42). While the relationship between IRIS and gut dysfunction in PLWH is still being studied, there are case reports that detail this phenomenon, including one in which IRIS presented as UC in a PLWH (43), and another in which a patient with IRIS and a history of HIV/AIDS experienced GI problems (including edema and erythema of mucosa with numerous shallow ulcerations revealed during colonoscopy) 2 months after starting ART (44).

As gut dysbiosis became a well-documented feature of HIV infection, research has turned to the role of diet as a means of restoring gut function and reducing inflammation. Though the mechanisms and efficacy of nutritional intervention are still being evaluated, it has been shown that poor dietary habits in people living with HIV can compromise immune function, increase comorbidities, and reduce survival and quality of life (45).

### 3.3 Using animal models to investigate gut barrier dysfunction and microbiota alterations in HIV and IBD

Pathogenesis of inflammatory gut dysfunction and associated diseases is complex and often involves multiple organ systems. The use of animal models to elucidate the intricacies of these interactions and evaluate the efficacy of pharmaceutical interventions is critical in advancing this area of research and developing novel drug solutions (46). For our study, animal models are also extremely useful in evaluating the effects of dietary interventions. Evaluating the effects of diet on disease progression can be complicated in human models due to confounding factors and ethical implications, issues that can be overcome by using animal models (47). Research using animal models has greatly contributed to our current understanding of nutrition and metabolism (48). However, while animal models are critical for analyzing disease pathogenesis and therapeutic interventions, they cannot perfectly reproduce human diversity or the epidemiological features of HIV infection (49).

A diverse range of animal models is key to understanding the pathogenesis and management of IBD. Mice and rats are often used for their practicality. Other animals, such as guinea pigs, New Zealand rabbits, pigs, zebrafish, *Drosophila melanogaster*, and nematodes, are also used for their unique physiologies (50). Notably, nonhuman primates (NHPs) serve as an excellent model for IBD, given their similarities to human pathology (51). For example, macaques frequently exhibit spontaneous colitis, which mirrors human IBD symptoms (52, 53), and diet may alter mucosal permeability in NHPs (47). IBD in animal models is measured using multiple factors, including clinical observation, the Disease Activity Index score, pathological observation, intestinal epithelial barrier integrity and fibrosis, inflammatory markers, and the intestinal microbiome. Though animal models cannot perfectly replicate human pathology, they are enormously useful in aiding our understanding of this complex disease (50). Experimental animal models have also provided important insights into the mechanisms linking intestinal barrier dysfunction, microbial dysbiosis, and chronic inflammation. Chemically induced models, particularly dextran sulfate sodium (DSS)-induced colitis, have demonstrated that epithelial injury can disrupt mucosal barrier integrity and trigger inflammatory responses resembling those observed in human IBD (54). These models are especially useful for studying the early events of disease development, including increased intestinal permeability, immune activation, and alterations in microbial communities. In addition, genetically engineered models have highlighted the importance of host–microbiota interactions in disease pathogenesis. For example, IL-10 knockout mice develop spontaneous intestinal inflammation in the presence of intestinal microorganisms (55), whereas disease severity is markedly reduced under germ-free conditions, emphasizing the critical role of the microbiome in maintaining intestinal homeostasis (56). Other experimental systems, including T-cell transfer models, have further contributed to understanding the immunological mechanisms responsible for chronic intestinal inflammation and barrier disruption (57). Together, these models provide valuable mechanistic insights that complement observations from human studies and support the development of targeted therapeutic and dietary interventions.

As in IBD, animal models, particularly NHPs, are similarly important in understanding the pathogenesis of HIV (58–63). Many African NHP species are naturally infected with species-specific simian immunodeficiency viruses (SIVs). Although they experience high levels of viral replication and CD4^+^ T-cell loss (particularly in the gut), natural hosts of SIV do not progress to AIDS (64). Comparing the differences in host mechanisms of NHPs that control SIV infection and those that progress to AIDS upon the SIV infection and/or PLWH may enhance our understanding of HIV infection and pave the way for new therapeutics and clinical interventions (65, 66). For example, natural hosts of SIV are particularly useful as animal models for understanding gut dysfunction in people living with HIV (29, 67). Unlike progressing hosts (humans and RMs), natural hosts of SIV maintain the integrity of the mucosal barrier throughout infection (68–70), do not show microbial translocation (71, 72), and do not experience chronic inflammation and T-cell immune activation (71–74). Therefore, studying how and why the gut is or is not impacted by infection in natural hosts and progressing hosts can teach us a great deal about disease progression (64, 75).

Altogether, our understanding of IBD and HIV is greatly enhanced using animal models. Their utility for studying gut dysfunction, microbiota alterations, and dietary interventions is highly relevant to this paper. Therefore, we will be integrating animal studies into our analysis.

### 3.4 Coexistence of HIV and IBD: a rare but intriguing clinical association

Although HIV infection and IBD are traditionally considered distinct clinical entities, several case reports and cohort studies have described patients with concomitant HIV and IBD, particularly UC. These cases provide a unique opportunity to examine the interaction between systemic immunodeficiency, intestinal inflammation, and gut microbial dysbiosis. Interestingly, some reports suggest that severe HIV-associated CD4^+^ T-cell depletion may be accompanied by reduced IBD activity, whereas intestinal inflammation can re-emerge following immune reconstitution after initiation of ART. For example, a patient with CD remained clinically stable during advanced HIV infection but experienced recurrence of gastrointestinal symptoms when CD4^+^ T-cell counts increased after ART, with remission being achieved through vedolizumab therapy (76). Other reports have highlighted the considerable management challenges posed by the coexistence of HIV and IBD, including an increased susceptibility to opportunistic infections, thromboembolic complications, and difficulties in balancing immunosuppressive therapy with the risk of further immune suppression (77).

Additional evidence supports the observation that HIV-associated immune dysregulation may impact IBD natural history. In a case report of new-onset UC in a woman with long-standing poorly controlled HIV infection, the authors noted that HIV may paradoxically exert a protective effect against IBD development and relapse through CD4^+^ T-cell depletion, which is central to intestinal inflammation. They proposed that substantial CD4^+^ T-cell loss within gut-associated lymphoid tissue (GALT) may attenuate the mucosal immune responses that drive IBD pathogenesis, a concept referred to as the “CD4 remission hypothesis” (78). Consistent with this hypothesis, other studies have reported lower relapse rates and milder disease courses among PLWH with IBD, particularly when CD4^+^ T-cell counts are below 200 cells/μL (79).

Meanwhile, diagnosing IBD in PLWH remains challenging because a variety of opportunistic and sexually transmitted infections (STIs) can closely mimic IBD both clinically and histologically. In a retrospective cohort of 50 PLWH with IBD, Siwak et al. found that UC accounted for 82% of cases and that only 20% experienced disease relapses during follow-up. The authors emphasized that infectious proctitis caused by pathogens such as *Chlamydia trachomatis*, *Neisseria gonorrhoeae*, herpes simplex virus, or *Treponema pallidum* may present with diarrhea, rectal bleeding, tenesmus, endoscopic ulceration, and inflammatory histopathological findings indistinguishable from IBD. Consequently, the authors recommended comprehensive HIV and STI screening, including molecular testing, in patients presenting with colorectal inflammation to avoid misdiagnosis and inappropriate immunosuppressive treatment (80). These observations further underscore the complex interplay between HIV infection, mucosal immunity, microbial exposure, and intestinal inflammation.

## 4 Gut barrier dysfunction and microbiota alterations as a common feature of HIV and IBD

Both HIV and IBD cause gut barrier disruption, but via different mechanisms. In HIV infection, the virus rapidly depletes mucosal CD4^+^ T cells (especially Th17 cells), weakening epithelial defense (81). This leads to “leaky gut” – tight-junction loss, microbial translocation, and systemic inflammation (30, 82, 83). In IBD (both CD and UC), chronic immune attacks on the gut lining produce ulcers and tight-junction defects (3). Ulceration and immune-cell infiltration in IBD markedly increase permeability. In both conditions, impaired mucus and epithelial integrity result in higher circulating lipopolysaccharides (LPS) and acute-phase markers (3, 84). Importantly, pediatric cases follow a similar pattern in both conditions: vertically HIV-infected children show early mucosal CD4^+^ T-cell loss and dysbiosis (85), while children with CD often require exclusive enteral nutrition to restore mucosal healing (86).

Dysbioses observed during both HIV infection and IBD are overlapping, yet distinct. HIV infection – especially untreated – is associated with markedly reduced bacterial diversity and an overgrowth of potentially inflammatory taxa (e.g., Proteobacteria, *Prevotella*) (82). IBD patients also have low diversity and loss of beneficial Firmicutes (notably *Faecalibacterium prausnitzii*), with relative Proteobacteria overabundance (87). In both diseases, the gut flora shifts away from fiber-fermenting commensals to “pathobionts,” fueling local inflammation and epithelial damage.

Beyond these compositional shifts in the gut microbiota, both HIV infection and IBD are also characterized by profound functional reprogramming of mucosal immune responses, particularly within the Th17/IL-22 axis, which critically determines epithelial barrier integrity. Physiological, nonpathogenic Th17 cells contribute to the maintenance of intestinal barrier maintenance through the production of IL-17A and IL-22, cytokines that stimulate epithelial tight-junction integrity, antimicrobial peptide secretion, mucus production, and epithelial regeneration (88). These protective functions are shared with Th22 cells and group 3 innate lymphoid cells (ILC3s), which together form an important immune–epithelial communication network that preserves the structure and function of the gut barrier (89). IL-22 in particular promotes epithelial proliferation and repair (90), whereas IL-17A enhances antimicrobial defenses and neutrophil recruitment, thereby limiting microbial invasion (91).

In IBD, however, Th17 responses become functionally dysregulated. Pathogenic Th17 cells, characterized by increased production of inflammatory mediators such as GM-CSF, IFN-*γ*, TNF-*α*, and IL-17A in an inflammatory cytokine milieu, contribute to chronic tissue injury and sustained leukocyte recruitment (92). This has led to the concept that Th17 cells display considerable plasticity, with protective and pathogenic phenotypes coexisting within the intestinal mucosa. Consequently, IL-17A effects may be contextual: while it supports epithelial barrier integrity under physiological conditions, excessive or dysregulated IL-17A signaling can amplify intestinal inflammation (93). This dual role may explain why therapeutic IL-17 blockade has limited efficacy and, in some cases, can worsen the outcome of CD (94, 95).

In HIV infection, the balance between pathogenic and nonpathogenic Th17 responses is profoundly disrupted, contributing to loss of intestinal immune–epithelial homeostasis. A hallmark of progressive disease is the early and preferential depletion of Th17 cells from gut-associated lymphoid tissue, accompanied by reduced IL-17A and IL-22 production, which weakens epithelial barrier integrity and promotes microbial translocation into systemic circulation (96). Concomitant impairment of Th22 cells and ILC3 further reduces IL-22 availability, thereby limiting epithelial regeneration and amplifying barrier damage (97). Furthermore, residual Th17 cells may undergo functional plasticity when exposed to inflammatory cytokines such as IL-1β, IL-6, and IL-23, acquiring Th1-like features such as increased IFN-*γ* and TNF-*α* production, with the final effect of shifting IL-17A signaling from a homeostatic to a pro-inflammatory role that may exacerbate mucosal injury and immune activation (30, 98). Collectively, these alterations establish a feed-forward loop of epithelial dysfunction, microbial translocation, and chronic immune activation that is central to HIV pathogenesis even on ART (99).

Table 1 summarizes key parallels and differences between the two clinical conditions.

**Table 1:** Comparison of immune cell dynamics, barrier integrity, and microbial alterations between HIV infection and IBD.

Features	HIV infection	IBD (CD/UC)	Impact on outcome/treatment approach
CD4^+^ T-cells	✓ Profound early loss in the GALT leads to leaky gut and systemic T-cell activation and depletion, and inflammation (98)	✓ Lymphocyte recruitment, but local CD4^+^ T-cells/Th17 may be high (177)	✓ Patients with IBD and concurrent HIV infection demonstrate a higher rate of remission compared with HIV-negative controls, which is associated with reduced total peripheral CD4^+^ T-cell counts (178)
CD8^+^ T-cells	✓ Increase of perforin-expressing mucosal CD8^+^ T cells during acute HIV infection, correlated with increased epithelial apoptosis (179) ✓ Colon tissue-resident memory (TRM) CD8^+^ T cells downregulate the lipid sensor peroxisome proliferator-activated receptor-γ (PPARγ) in PLWH on ART leading to impaired cellular lipid metabolism, intestinal epithelial cell apoptosis and barrier damage (180)	✓ Cytotoxic CD8^+^ T cells (Tc1) and IL17-producing CD8^+^ T cells (Tc17) are proinflammatory and contribute to IBD pathogenesis (181) ✓ CD103^+^ TRM cells support mucosal homeostasis, while pro-inflammatory CD103^+^ TRM (granzyme K^+^, Th17-related) and KLRG1^+^ TRM subsets are enriched in inflamed tissue and display enhanced cytotoxicity (181)	✓ In PLWH on ART, colon TRM CD8^+^ T cells exhibit lipid droplet depletion and impaired fatty acid oxidation, reflecting disrupted metabolic homeostasis that can be restored by PPARγ agonism (rosiglitazone) (180) ✓ Reprogramming pro-inflammatory TRM cells toward a tolerogenic phenotype (e.g., through continuous antigen exposure) may represent a potential therapeutic strategy in IBD (181)
Barrier integrity	Disrupted – loosen tight junctions; ↑ microbial translocation (182)	Ulceration/erosions and chronic leakage (183)	- Mucus layer restoration and inhibition of intestinal epithelium permeability by nutritional dietary fiber intake during remission, exogenous mucins during active phase, supplementing short-chain fatty acids (SCFAs) or pharmacological interventions (e.g., targeting occludin and claudin-2) in IBD (184)
Microbial diversity	↓ Diversity; ↑ Proteobacteria and often *Prevotella* (185)	↓ Diversity; ↑ Proteobacteria (e.g., *Enterobacteriaceae*) (186)	Microbiota-targeted interventions (dietary modulation, pre−/probiotics, postbiotics) may improve outcomes in IBD; in HIV, these strategies may complement ART by reducing microbial translocation and chronic immune activation
Beneficial taxa	Loss of SCFA-producers (e.g. *Bifidobacterium*, *Clostridia*) (187)	Loss of *F. prausnitzii*, increased pro-inflammatory *Ruminococcus* (188)	- Therapeutic strategies aimed at restoring beneficial taxa or their metabolites (e.g., high-fiber diets, targeted probiotics, postbiotic SCFAs, or microbiota-based therapies) may enhance barrier integrity, promote immune tolerance, and improve disease outcomes (104, 189)

ART, antiretroviral therapy; CD, Crohn’s disease; GALT, gut-associated lymphoid tissue; HIV, human immunodeficiency virus; IBD, inflammatory bowel disease; IL-17, interleukin 17; KLRG1, killer cell lectin-like receptor subfamily G member 1; LPS, lipopolysaccharide; PLWH, people living with HIV; PPARγ, peroxisome proliferator-activated receptor gamma; SCFA, short-chain fatty acid; Tc1, type 1 cytotoxic T cells; Tc17, interleukin-17-producing cytotoxic T cells; Th17, T helper 17 cells; TJ, tight junction; TRM, tissue-resident memory T cells; UC, ulcerative colitis.

## 5 Beneficial dietary/nutritional interventions for alleviating IBD and HIV infection

### 5.1 High-fiber diets

Fiber fermentation yields short-chain fatty acids (SCFAs), such as butyrate and propionate, which are key colonocyte fuels and provide anti-inflammatory signals. Butyrate supports epithelial cells metabolism and contributes to the restoration of tight junctions integrity (100, 101). SCFAs upregulate barrier proteins (occludin, claudins) and induce regulatory T cells (Tregs) via TGF-*β*/retinoic acid pathways, shifting immunity toward tolerance (102). Through these effects, high-fiber, plant-rich diets increase microbial diversity and SCFA production, which may help both HIV and IBD patients maintain gut integrity and suppress inflammation (103). Nevertheless, not all interventions targeting SCFAs lead to beneficial effects. For instance, oral administration of sodium butyrate was insufficient to reduce persistent inflammation and microbial translocation in ARV-treated, SIV-infected macaques, and did not significantly improve immune reconstitution, with no differences observed in systemic CD4^+^ T-cell frequencies, T-cell functionality or immune activation, microbial translocation, or transcriptional regulation (104).

### 5.2 Mediterranean diet

A diet rich in fruits, vegetables, whole grains, and omega-3 fats reduces oxidative stress and systemic inflammation. In adult PLWH, adherence to a supplemented Mediterranean diet improved immune activation markers, Treg function, and gut microbiota composition (105). In patients with IBD (including children), Mediterranean-style eating lowered the levels of C-reactive protein (CRP) and fecal calprotectin and enhanced SCFA production (106). For example, in a pediatric ulcerative colitis trial, children on a Mediterranean regimen had greater drops in calprotectin and inflammatory cytokines than controls (107). Mucin 2-deficient mice (which develop spontaneous colitis) fed a Mediterranean diet fat blend were protected from developing severe colitis, and showed decreased inflammation-related biomarkers (108). The Mediterranean diet is also a major source of bioactive compounds, particularly dietary polyphenols (e.g., curcumin, resveratrol, hydroxytyrosol), which exert antioxidant and immunomodulatory effects (109). These compounds contribute to the modulation of gut microbiota, enhancement of epithelial barrier integrity, and down-regulation of pro-inflammatory pathways (109). Similar anti-inflammatory and microbiome-mediated benefits have been described in both IBD (110) and PLWH (111), conditions characterized by chronic immune activation and intestinal dysbiosis (112, 113).

### 5.3 Vitamin A (retinol) and carotenoids

Vitamin A is essential for mucosal health. It promotes mucus secretion and epithelial integrity, as suggested by the observation that vitamin A deficiency leads to keratinization and barrier thinning (114). Immunologically, the active metabolite all-trans retinoic acid (ATRA) inhibits inflammatory Th17 differentiation and enhances FoxP3^+^ Treg induction (115). Thus, optimal levels of vitamin A help maintaining a robust mucosal barrier and steer immunity toward regulation. However, the role of vitamin A in HIV infection appears more complex than its generally protective effects on mucosal immunity. While ATRA supports epithelial integrity and immune regulation, it can also increase HIV permissiveness in CCR6^+^ T cells and macrophages, potentially enhancing viral transcription and replication (116, 117). More specifically, ATRA is linked to increased CCR5 expression on these cells, facilitating viral entry, and promoting HIV replication through postentry mechanisms (116, 118). Clinical trials evaluating vitamin A supplementation in PLWH have yielded inconsistent results, suggesting that its immunological effects may be context-dependent (119). Moreover, the vitamin A derivative acitretin, widely used in psoriasis treatment, has been investigated as a latency-reversing agent capable of promoting HIV reservoir reactivation (120). Retinoic acid supplementation may enhance reactivation of competent HIV/SIV reservoirs, having therapeutic potential as a “shock and kill” approach to HIV reservoir depletion (121). Retinoic acid also induces the expression of the gut-homing integrin α4β7 on lymphocytes, facilitating their migration to the intestine (122). This pathway is particularly relevant in IBD, where α4β7 blockade with Vedolizumab reduces intestinal leukocyte trafficking and inflammation (123). Collectively, these findings indicate that, while vitamin A contributes to mucosal barrier maintenance and immune regulation, its effects on HIV pathogenesis and intestinal immunity are multifaceted, warranting cautious interpretation when considering its therapeutic implications in either HIV infection or IBD (Table 2).

**Table 2:** Dietary/nutritional factors – mechanisms and effects in HIV/IBD.

Factor	Mechanism of action	Effect	Evidence
High-fiber diet (↑SCFAs^1^)	Fiber → fermentation → butyrate/propionate → colonocyte fuel; ↑ TJ proteins and Tregs (190)	Beneficial (strengthens barrier, anti-inflammatory)	Recognized protection in IBD and support for gut health in PLWH (191, 192)
Mediterranean diet	High antioxidants, *ω*-3 fats; ↑microbial diversity and SCFAs (193)	Beneficial (lowers inflammation)	Improves immune activation in HIV, lowers calprotectin in IBD (194, 195)
Vitamin A	Promotes mucin secretion; maintains epithelial maturation; ATRA inhibits Th17/promotes Treg (196)	Beneficial (improves mucosal integrity, immune regulation)	Vitamin A therapy enhances barrier and shifts T cells programming to an anti-inflammatory phenotype in animal models (197)
PPARγ agonists (ω-3, butyrate)	Activates PPARγ: ↑*β*-oxidation in T cells, M2 macrophages; ↑goblet cells & tight junctions (198)	Beneficial (anti-inflammatory, barrier-enhancing)	PPARγ ligands/THC improve gut integrity in HIV/colitis models (124, 199)
Predigested protein (enteral)	Nutrient formula → supports epithelial repair; supplies amino acids	Beneficial (supports CD4^+^ T-cell recovery, gut healing)	↑CD4 counts and ↓ gut leak markers in HIV and IBD (128, 200)
Omega-3 fatty acids	Altered gut microbiota; ↑ production of anti-inflammatory compounds (129)	Beneficial (strengthens barrier, reduces inflammation)	↓ in inflammation and gut permeability in HIV. ↓ proinflammatory cytokines in IBD (130, 131)
High-fat/high-sugar diet (Western diet-WD)	↑ Pro-inflammatory cytokines; oxidative stress on enterocytes (136) tight junction (TJ) breakdown and dysbiosis (201)	Deleterious (increases permeability)	WD linked to IBD onset (202) exacerbates HIV-1 rectal transmission (203)
Excess fructose	Alters TJ proteins; ↑ Gram-negative bacteria; ↑ endotoxin translocation (204)	Deleterious (weakens barrier)	High fructose feeding increases gut leakage and plasma LPS in animal models (144)
Micronutrient deficits	Impaired mucus/immune function (e.g. vitamin A loss thins mucus) (205)	Deleterious (primes inflammation)	Low vitamin D correlates with ↑CRP in IBD; Vitamin A deficiency impairs barrier (206)
Alcohol consumption	Alters composition of gut microbiota; ↑ LPS translocation; ↑ Pro-inflammatory cytokines (207)	Deleterious (impairs gut integrity, changes composition of gut microbiota)	Alcohol alters gut integrity and ↑ inflammation and microbial translocation in PLWH (154, 208); ↑ symptoms and propensity for infection in IBD patients (157)

ART, antiretroviral therapy; ATRA, all-trans retinoic acid; CD, Crohn’s disease; CRP, C-reactive protein; GALT, gut-associated lymphoid tissue; HIV, human immunodeficiency virus; IBD, inflammatory bowel disease; IL, interleukin; KLRG1, killer cell lectin-like receptor subfamily G member 1; LPS, lipopolysaccharide; PPARγ, peroxisome proliferator-activated receptor gamma; PLWH, people living with HIV; SCFAs, short-chain fatty acids; Tc1, type 1 cytotoxic T cells; Tc17, IL-17-producing cytotoxic T cells; Th17, T helper 17 cells; TJ, tight junction; Treg, regulatory T cells; TRM, tissue-resident memory T cells; UC, ulcerative colitis; WD, Western diet; ω-3, omega-3 fatty acids.

### 5.4 Peroxisome proliferator-activated Receptor gamma (PPARγ) agonists

Activation of the nuclear receptor PPARγ in immune and epithelial cells shifts metabolism towards fatty-acid oxidation and suppresses NF-κB inflammation (124). In T cells and macrophages, PPARγ drives an anti-inflammatory, oxidative state (124). Nutrients like omega-3 fatty acids and butyrate are natural PPARγ ligands (125). Pharmacologic PPARγ activators (e.g., thiazolidinediones) and cannabinoids (e.g., tetrahydrocannabinol) have shown anti-inflammatory, barrier-protective effects in colitis and SIV (simian HIV) models (124). For instance, THC (a PPARγ agonist) improved gut integrity and reduced translocation in SIV-infected monkeys (126). Overall, PPARγ activation restores lipid homeostasis in immune cells and prevents epithelial apoptosis in both HIV and IBD contexts (124).

### 5.5 Enteral nutrition (especially in children)

Exclusive enteral nutrition (EEN) is the first-choice therapeutic approach aimed at inducing remission and promoting mucosal healing in pediatric Crohn’s disease (127). EEN induces remission as effectively as steroids while promoting mucosal healing, nutrition, and growth (86). It works partly by dramatically altering the microbiome (increasing diversity and reducing pathogens) (87). In PLWH who are “immunological non-responders (INR)” (poor CD4^+^ T-cell recovery on ART), specialized pre-digested protein supplements have likewise improved gut outcomes. In a study (128) evaluating the effects of a pre-digested enteral nutritional supplement in INR PLWH over a three-month period, supplementation significantly improved immune recovery, as shown by increased CD4^+^ and CD8^+^ T-cell counts (128). Markers of intestinal mucosal damage (DAO, D-lactate, and LPS) were markedly reduced, indicating improved gut integrity. Nutritional status also improved, with significant gains in body weight, body mass index (BMI), albumin, and hemoglobin (128). Correlation analysis revealed that higher CD4^+^ T-cell counts were associated with lower intestinal damage markers, while elevated IL-1β levels were strongly linked to increased gut permeability markers. Overall, the findings demonstrate that predigested enteral nutrition effectively enhances immune function, gut barrier integrity, and nutritional status in HIV-infected INRs (128). Thus, tailored nutritional therapy can aid gut repair and immune reconstitution in both pediatric IBD and PLWH, particularly INRs.

### 5.6 Omega-3 fatty acid

Though high-fat diets are shown to promote gut dysbiosis and permeability, there are some dietary fats, like omega-3 polyunsaturated fatty acids, which can have positive effects on the intestinal environment. More specifically, it has been found that supplementation with omega-3 polyunsaturated fatty acids has the capability of restoring eubiosis in the gut microbiota (by restoring the *Firmicutes/Bacteroidetes* ratio and increasing *Lachnospiraceae* taxa) and increasing the production of anti-inflammatory compounds, like short-chain fatty acids (129). In PLWH, 12 weeks of fish oil supplementation reduced inflammation and gut permeability (130). Similarly, in patients with IBD, omega-3 fatty acids are shown to reduce intestinal inflammation, associate with the reduction of proinflammatory cytokines, decrease disease activity, and improve quality of life (131). One study showed that transgenic mice rich in endogenous omega-3 fatty acids had reduced inflammation and tissue injury in colitis (132).

## 6 Detrimental dietary approaches

### 6.1 High-fat/high-sugar (“Western”) diet

The modern high-sugar and high-fat diet—frequently termed the “Western diet”—is no longer viewed merely as a source of excess calories. Instead, it is recognized as a potent, noninfectious disruptor of human physiology (133). When consumed by individuals with underlying chronic inflammatory conditions like HIV or IBD, a high-sugar diet acts as a universal accelerant (134). It triggers a cascade of cellular stress, shifts the gut microbiome, destroys the intestinal lining, and ultimately drives the development of glucose intolerance and Type 2 Diabetes (T2D) (135). Diets rich in saturated fat, simple sugars, and processed foods promote gut permeability and dysbiosis. Moreover, excess fat triggers the release of proinflammatory cytokines (TNF-*α*, IL-1β, IL-6) and oxidative stress in enterocytes, loosening tight junctions (136). It also favors bile acids and microbes that further disrupt the mucus barrier (136). In IBD epidemiology, Western diets (red meat, refined carbs, high fat) correlate with higher IBD risk, whereas fiber-rich diets are protective (87). In PLWH, similar Western diets likely exacerbate microbial translocation and immune activation, although direct trials are limited (137). In both nonpathogenic and pathogenic hosts of SIV, a HFD negatively impacted SIV progression and survival. This includes increased Inflammation and immune response, immune cell infiltration in the adipose tissue, alteration of the intestinal immune environment, gut damage, and microbial translocation. In a nonpathogenic model (AGMs), a HFD induced progression to AIDS (47). Overall, a high-fat/sugar diet is detrimental – it drives a “leaky gut” and outgrowth of proinflammatory pathobionts, ultimately leading to intestinal inflammation, metabolic dysfunction, and systemic immune activation. Consistent with this concept, growing evidence indicates that metabolic disorders such as T2D are closely interconnected with chronic inflammatory diseases, including both IBD and HIV infection, through shared mechanisms involving gut barrier dysfunction, chronic inflammation, and immune dysregulation. In animal models, diabetes aggravated colitis severity in high-fat diet–fed mice by impairing intestinal barrier integrity, an effect that was reversed after restoration of normoglycemia (138). In humans, the link between IBD and T2D proved to be bidirectional: patients with IBD had a 44% increased risk of developing T2D, while patients with T2D had a 40% increased risk of developing IBD. Moreover, greater disease severity in either condition strengthened this association, supporting shared inflammatory and metabolic mechanisms underlying both disorders (139). Furthermore, the coexistence of T2D and IBD has been associated with higher rates of hepatic steatosis and liver damage (140). Interestingly, UC accounted for approximately 71–74% of IBD cases among patients with concomitant T2D, suggesting a stronger clinical and immunological association between UC and T2D than between Crohn’s disease and T2D and further supporting the concept of shared immune-mediated inflammatory pathways linking intestinal and metabolic disease (141). The common feature of both these diseases is the chronic systemic inflammation, which is also the main determinant of disease progression in HIV infection. Proinflammatory cytokines, including TNF-*α* and IL-6, contribute to insulin resistance, a central feature of T2D, while dysregulated intestinal immune responses in IBD may amplify systemic inflammation and promote metabolic dysfunction (141).

A similar relationship has been observed between HIV infection and T2D. As life expectancy among PLWH has increased with the widespread use of ART, metabolic comorbidities such as insulin resistance, obesity, metabolic syndrome, and T2D have emerged as major contributors to long-term morbidity (142). Beyond their metabolic consequences, these conditions may also accelerate immune aging. The coexistence of chronic HIV infection with insulin resistance and MASLD (Metabolic dysfunction-Associated Steatotic Liver Disease) promotes a state of persistent low-grade inflammation (“metaflammation”), characterized by gut dysbiosis, microbial translocation, and hepatic inflammation (143). Activation of hepatic PRRs and the NLRP3 inflammasome contributes to mitochondrial dysfunction and the accumulation of senescent CD8^+^ CD28^neg^ T cells, ultimately driving immunosenescence. In this context, the Triglyceride-Glucose (TyG) index, a surrogate marker of insulin resistance, may reflect the immunometabolic disturbances linking metabolic dysfunction to accelerated immune aging in PLWH (143).

### 6.2 Excess fructose

High intake of fructose (e.g., sugary drinks) has been shown to damage the gut barrier. In rodent models, fructose diets increase intestinal permeability and plasma endotoxin levels (144). Fructose alters tight-junction proteins and shifts gut microbiome toward endotoxin-producing flora (145). High fructose consumption has also been associated with gut microbial dysbiosis characterized by reduced microbial diversity and depletion of beneficial butyrate-producing bacteria, including *Faecalibacterium*, *Ruminococcus*, *Ruminococcaceae*, and *Lachnospiraceae* (146). This finding is particularly relevant because reduced abundance of butyrate-producing bacteria has been linked to both IBD and T2D (147). Butyrate plays a key role in maintaining intestinal barrier integrity through AMPK-dependent tight-junction assembly and exerts anti-inflammatory effects by suppressing NF-κB signaling (148, 149). As a result, fructose-induced reductions in butyrate-producing bacteria may further impair mucosal barrier function and promote intestinal inflammation. Increased fructose availability has also been linked to MetS, and is associated with an increased incidence of diabetes. In mice and rats, fructose administration induced obesity, metabolic syndrome, diabetes, and fatty liver (150). PLWH are at an increased risk of developing T2D, not only due to lifestyle and dietary practices, but also because some antiretrovirals can trigger to metabolic disorders (like insulin resistance) and affect glucose regulation. As ART has greatly increased the lifespan of PLWH, comorbidities such as diabetes have become a focus for improving health outcomes for these individuals (151).

### 6.3 Micronutrient (vitamins/minerals) deficiencies

Chronic HIV and IBD often entail deficiencies of iron, zinc, and fat-soluble vitamins (A, D, E, K). These deficits prime the immune system for inflammation. For example, vitamin A deficiency was shown to thin the intestinal mucosa and impair barrier defenses (115). Vitamin D insufficiency, which is a common clinical condition in patients with IBD and PLWH, correlates with higher levels of systemic inflammation (i.e., increased CRP levels) and more severe disease (152). Likewise, iron deficiency impairs immune homeostasis and can itself drive an acute-phase response (elevated CRP) (153). In sum, lack of key nutrients worsens mucosal integrity and skews toward chronic inflammation. Correcting these deficiencies (e.g., supplementing vitamins A/D/E) is therefore beneficial, whereas allowing them to persist contributes to disease flares.

### 6.4 Alcohol consumption

Heavy consumption of alcohol is known to promote systemic inflammation and impair gut integrity. In PLWH, alcohol consumption is associated with increased microbial translocation and accelerated disease progression (154). Understanding the effects of chronic alcohol use on HIV progression is significant, given that rates of heavy drinking in PLWH are almost twice those found in the general population (155). Similarly, in individuals living with IBD, heavy alcohol consumption is associated with inflammation and poor IBD outcomes. Thus, patients with IBD report worse GI symptoms after consuming alcohol (156). In mice that underwent a binge alcohol paradigm followed by induced colitis, ulcerative colitis symptoms increased, and propensity to infection was heightened (157).

## 7 Food insecurity

As diet plays a significant role in the pathogenesis of both HIV and IBD, it is important to consider the social factors that limit access to adequate nutrition.

Food insecurity is particularly prevalent among PLWH, with approximately a quarter to a half of PLWH in the United States being affected, compared to just 14% of the general population (158).

In people living with HIV, factors like gender, poverty, and drug use are all correlated with limited access to food (159). Regarding disease pathogenesis, food insecurity is associated with worse HIV outcomes, including lower CD4^+^ T-cell counts, incomplete viral load suppression, and decreased survival. ART nonadherence is also elevated in these individuals, compromising HIV management (159, 160). The relationship between HIV/AIDS and food insecurity has been described as a “vicious cycle,” in which food insecurity worsens HIV outcomes and enhances HIV transmission, while the social stigma of the disease and economic consequences of treatment drive food insecurity. Current approaches to dietary intervention are limited, and a robust response requires collaboration from many sectors, including government, healthcare, and agriculture (161).

Individuals living with IBD are similarly affected by food insecurity. In a cohort of 128 individuals diagnosed with IBD, 36% were diagnosed with malnutrition, and 48% were identified as “at risk for food insecurity,” based on the Hunger Vital Sign™ recommended scoring. Notably, at-risk patients were also more likely to consume ultra-processed foods and less likely to consume unprocessed foods than those who were not at-risk (162). Furthermore, individuals with IBD are nearly twice as likely to experience food insecurity compared to the average US adult. Insufficient access to food puts individuals with IBD at risk for nutritional deficiencies and can heighten GI symptoms (163). Overconsumption of ultra-processed foods has been independently linked to increased GI illness and heightened risk of developing IBD (162). It is therefore recommended that patients with IBD be screened for food insecurity throughout their treatment (162).

Food insecurity significantly impacts health outcomes and is associated with increased inflammation, a defining feature of both HIV and IBD pathogenesis (164). Accordingly, allocating time and resources towards combating food insecurity can be a powerful tool in mitigating disease progression and potentially reducing new cases.

## 8 Microbiota-targeted therapies: fecal microbiota transplantation

Fecal microbiota transplantation (FMT) represents a microbiome-driven therapeutic strategy aimed at restoring microbial diversity and intestinal homeostasis through the transfer of fecal material from healthy donors to affected recipients (165). FMT has demonstrated remarkable efficacy in recurrent *Clostridioides difficile* infection (166) and has subsequently been investigated in chronic inflammatory disorders characterized by dysbiosis, including IBD and HIV infection.

In UC, FMT can induce clinical remission and endoscopic improvement in a subset of patients, particularly when intensive multidonor protocols are used (167). These benefits are associated with increased microbial diversity, enrichment of short-chain fatty acid–producing bacteria, and restoration of metabolic pathways involved in epithelial barrier maintenance (168). However, responses to this therapeutic strategy varies widely, and long-term efficacy has yet to be fully established. Evidence in CD is more limited, although preliminary studies suggest potential improvements in disease activity and microbial composition (169). Thus, while FMT did not significantly improve endoscopic remission, it resulted in higher rates of clinical remission at eight weeks compared to placebo (170). Meanwhile, repeated FMT administrations were more effective than single-dose treatments (171). Additionally, FMT led to improvements in the CD Endoscopic Index of Severity (CDEIS) and reductions in CRP levels compared to placebo (172).

In HIV infection, FMT remains largely investigational. Early clinical studies reported that FMT is safe and can partially modify gut microbial composition in PLWH by increasing bacterial diversity and the abundance of beneficial commensals (173). In an open-label study involving ART-treated, virologically suppressed individuals,donor microbial engraftment occurred after FMT, although it was modest and limited to specific bacterial taxa (174). Despite these microbiome changes, no significant reductions in systemic inflammatory markers were observed during follow-up, suggesting that greater microbial engraftment may be required to achieve meaningful immunological benefits (174).

Taken together, these findings support the potential role of FMT as an adjunctive strategy for correcting dysbiosis and restoring gut homeostasis, particularly in IBD. However, additional studies are needed to optimize donor selection, treatment protocols, durability of response, and safety, especially in immunocompromised populations. Due to these limitations, and to the clinical risks associated to microbial transplantation, dietary interventions, which have proven effects on microbiome and can provide more natural changes in the microbiome composition, appear to be a strategy of choice to this goal.

## 9 Conclusion

The relationship between chronic immune activation and gut dysfunction is well-established and is critical for understanding the pathogenesis of diseases like HIV and IBD. Understanding the progression of these complex diseases is aided by using animal studies, which help eliminate confounding factors and, in the case of natural hosts of SIV, teach us a great deal about disease mechanics.

As we have shown, dietary factors, both beneficial and harmful, have a significant impact on disease progression. Encouraging people living with HIV and IBD to follow anti-inflammatory diets (like a Mediterranean or high fiber diet) or supplement their diet with certain nutrients (like Vitamin A or Omega-3 fatty acids) is an excellent non-invasive approach for preventing or ameliorating gut dysfunction caused by chronic inflammation. However, providers must consider the impact of food insecurity on procuring nutritious food and work with affected patients to find assistance programs.

Studies focused on nutrition and the complexities of the gut microbiome are currently becoming increasingly relevant. This research is highly applicable to our model diseases, IBD and HIV. Though the exact mechanisms of gut dysfunction and the effects of diet are still not fully understood, nutritional assessment and individualized dietary plans should be integrated into the care plan for PLWH and people living with IBD. Dietary modulation should not be considered a substitute for the treatment of these diseases, but rather as a complement to clinical intervention, aimed at improving quality of life, reducing inflammation, reducing comorbidities, and improving clinical outcomes (47, 175, 176).
